# Rare occurrence of metastasis from lung cancer to the anus: case report and review of the literature

**DOI:** 10.1186/s12957-016-0909-2

**Published:** 2016-06-08

**Authors:** Mohannad Al-Tarakji, Jonas Feilchenfeldt, Abdulrazzaq Haidar, Lajos Szabados, Sherif Abdelaziem, Ali Sayed, Adriana Toro, Isidoro Di Carlo

**Affiliations:** Department of Surgery, Hamad General Hospital, Al Rayyan Road, 3050 Doha, Qatar; National Center for Cancer Care and Research, Doha, Qatar; Department of Pathology, Hamad General Hospital, Doha, Qatar; PET/CT Center, Clinical Imaging Department, Hamad General Hospital, Doha, Qatar; Department of Surgery, Patti Hospital, Messina, Italy; Department of Surgical Sciences and Advanced Technologies, “G.F. Ingrassia” University of Catania, Catania, Italy

**Keywords:** Lung, Metastases, Anal cancer

## Abstract

**Background:**

Anal metastases from lung cancer are infrequent, and there are only 10 published cases. Life expectancy is no longer than 1 year after diagnosis because of the typically advanced stage of disease. Treatment, which is typically inefficient, is administered with the intent to cure or avoid local complications.

**Case presentation:**

We report a case of a patient with non-small cell lung cancer presenting with perianal metastasis mimicking an abscess.

**Conclusions:**

Because perianal masses may be misdiagnosed, patients with lung and other cancers should be evaluated for metastatic disease.

## Background

It is very rare for metastases from lung cancers to reside in the anal and/or perianal regions or to associate with multiple metastases in other organs [1]. Accordingly, there are few published reports of this phenomenon. Life expectancy is very short in such cases because this localization represents the dissemination of an aggressive cancer [2]. We describe a patient with non-small cell lung cancer (NSCLC) presenting with perianal metastatic disease mimicking an abscess.

## Case presentation

A 75-year-old man presented on July 2014 with weight loss, loss of appetite, and fatigue. He was a heavy smoker who smoked one pack of cigarettes daily for 40 years. A chest X-ray revealed a homogenous opacity in the left-apical region. Computed tomography (CT) of the chest detected a mass in the left-apical segment (5.3 cm × 6 cm × 6.5 cm) with tiny focal calcifications within the mass, erosion of the left posterior region of the second rib, and multiple mediastinal lymph nodes <1 cm. Histopathological analysis led to the diagnosis of NSCLC consistent with adenocarcinoma.

A CT scan of the abdomen and pelvis did not detect metastasis, and magnetic resonance imaging did not detect intracranial metastases. Iron deficiency anemia (hemoglobin = 10.2 g/dL) was present. Positron emission tomography (PET) detected intense hypermetabolism in the known left-apical Pancoast tumor, which appeared to invade the thoracic intervertebral foramina with mediastinal and the left axillary lymph node, a suspicious C5 vertebral body metastasis, and intense uptake of tracer in the gastric wall, likely representing a malignancy. Magnetic resonance imaging of the spine detected a left-lung apical tumor with encroachment of the left supraclavicular region as well as extension into the left intervertebral DV1 and DV2 foramina. The tumor displaced, compressed, and invaded the adjacent trunks and distal roots of the brachial plexus with edema surrounding the root of the left neck, the ipsilateral brachial plexus, and the left shoulder with alteration of signal intensities of peri-shoulder muscular anatomy. Upper gastrointestinal endoscopy revealed status post Billroth 2 surgery with erosive gastritis without malignancy.

**Fig. 1:**
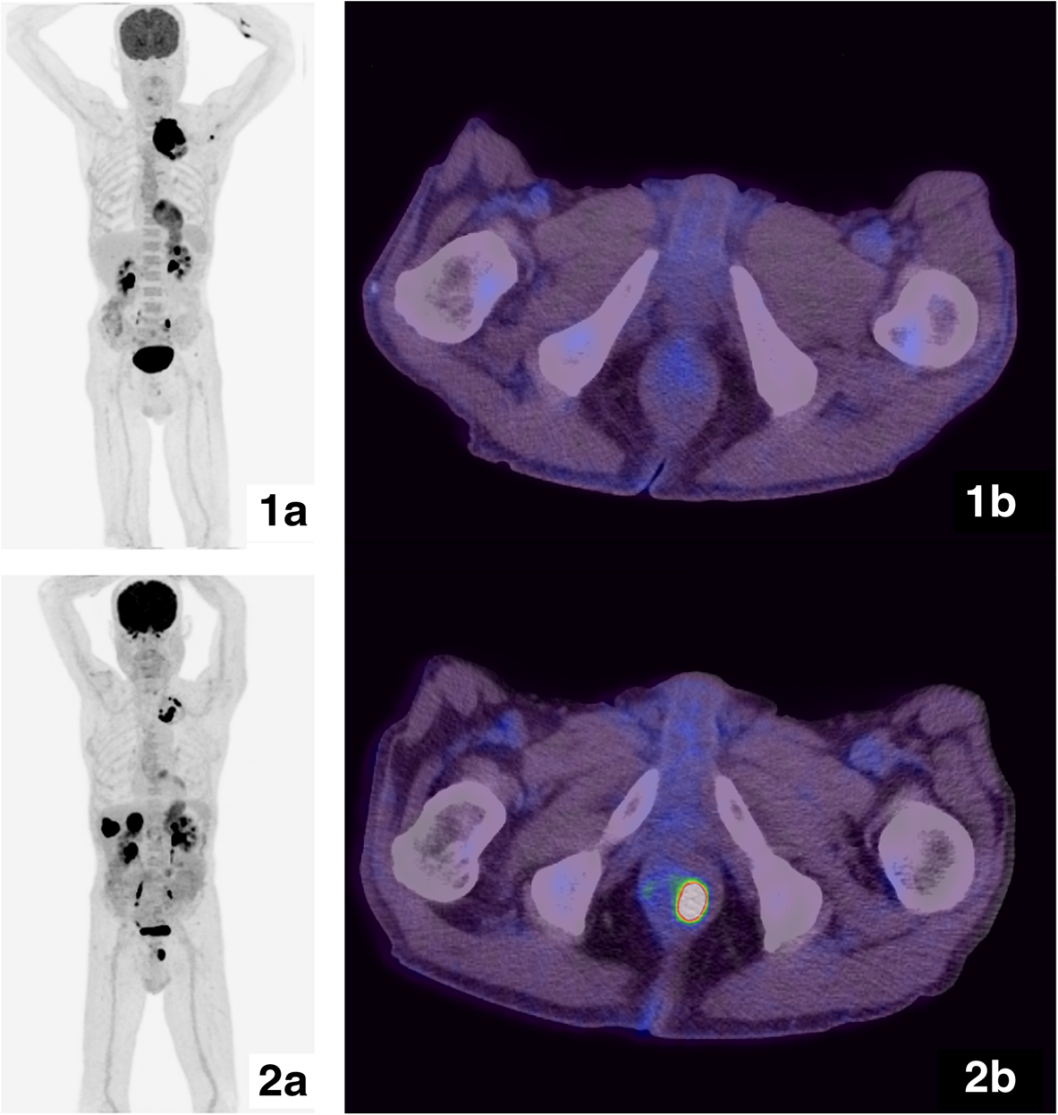
Maximum intensity projection (MIP) image of ^18^F-fluorodeoxyglucose-staging PET/CT showing hypermetabolic left-lung primary and mediastinal/left axillary lymph node involvement (**1a**). Transaxial superimposed PET/CT images showing no focal pathological uptake at the anus (**1b**). After four cycles of chemotherapy, the MIP image revealed new involvement of the adrenals, multiple bone metastases, and perianal uptake. Partial metabolic response in the left primary lung tumor (**2a**). Transaxial superimposed PET/CT images showing intense focal uptake at the left aspect of the anus (**2b**)

Palliative radiotherapy of the primary tumor was administered along with pemetrexed and carboplatin, and zoledronic acid was continued. In October 2014, the patient presented with pain and tenderness in the left lower chest wall. Ultrasonography revealed a slightly heterogeneous liver mass with small hyperechoic foci (<1 cm) within the right hepatic lobe and a heterogeneous hypoechoic lesion (4.6 × 3.1 cm) in the left adrenal. A CT scan of the chest showed a soft-tissue dense ovoid mass (approximately 3 cm × 4.5 cm along the right lower chest wall anterior and opposite the anterior ends of the seventh and eighth ribs). A PET scan revealed high tracer uptake into the newly developed bilateral adrenal metastasis and right chest wall lesion. Anal tracer uptake suggested metastasis, and we discontinued testing because the patient was asymptomatic (Fig. 1).

**Fig. 2:**
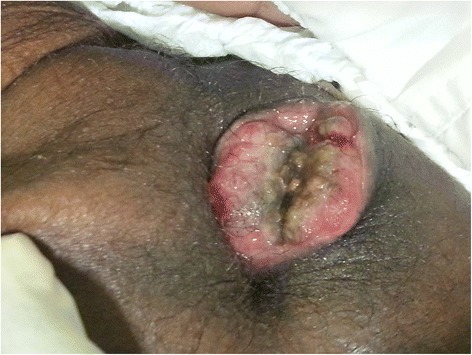
Anal metastases

**Fig. 3:**
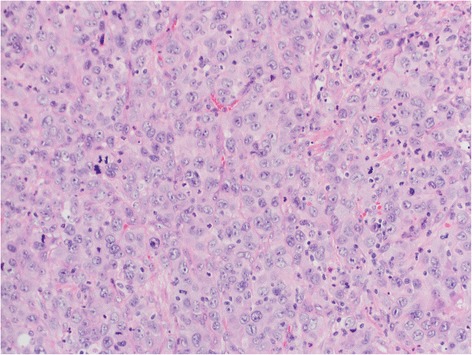
Histopathological analysis of the anal mass revealed poorly differentiated carcinoma comprising large round cells with abundant cytoplasm and large nuclei with one to two prominent nucleoli

The patient was referred for surgery in February 2015 with a complaint of severe perianal pain and involuntary defecation. A painful, fungating, and red to brown ulcerating mass (approximately 3 cm × 4 cm) was present near the anal orifice, which was covered by friable tissue with traces of stool (Fig. 2). Histopathology detected a metastatic, poorly differentiated carcinoma with histomorphology and immunohistochemical findings (CKAE1/AE3^+^, vimentin^+^) identical to those of the lung tumor (Fig. 3). The patient became severely cachectic and delirious and was short of breath. He died in March 2015 after receiving palliative care.

### Discussion

Lung cancer is the most frequent malignancy with a high mortality rate [3], and patients with NSCLC account for 80–85 %. Adenocarcinomas represent approximately 40 % of NSCLC [4]. When first diagnosed, approximately 40 % of patients with NSCLC harbor distant metastases, and the recurrence rate is high in patients with early disease [4]. Most metastatic lung cancers directly invade other organs and spread through the blood or lymph as well. The brain, bones, adrenal glands, and bone marrow are common metastatic sites of lung cancers compared with the gastrointestinal tract [2].

Autopsy findings indicate that the rate of gastrointestinal metastases from primary lung carcinomas ranges between 4.7 and 14 % [5]. The esophagus is commonly invaded by direct extension, whereas the stomach is invaded through the lymphatics. One study detected metastatic cells in the small and large bowels [5]. Anal cancers represent 1–2 % of gastrointestinal cancers in the USA; metastatic lesions are less frequent (http://www.fascrs.org/patients/conditions/analcancer/), and metastatic colon cancer and exfoliation cause most cases [6]. In contrast, metastasis to the intestine from the stomach to the anus is likely caused by retrograde spread through the lymphatics that connect the lung through the mediastine to the intestines [4]. Suspicious hematogenous spread, consequent to cardiopulmonary bypass circuit used to remove a lung tumor protruding into the atrium, has been reported [7]. However, we were unable to determine the route of metastasis in our patient.

There are 11 reports of perianal and anal metastases from primary lung cancers, including the present study (Table 1) [1, 6–14]. The 10 patients with NSCLC included five with adenocarcinomas. Anal metastases were metachronous in 8/11 patients; other metastatic sites were present, and an abscess was the most prevalent finding. Every abscess or fistula in at-risk patients, such as smokers, has to be considered as a potential metastatic tumor from the lung. Establishing a diagnosis must account for adenocarcinomas arising from a fistula associated with Crohn’s disease [15], an anal gland adenocarcinoma [16], or rare metastases from breast [17], pancreatic [18], or renal cancer [19]. Patients should be administered single or combined chemotherapy and radiotherapy, because surgery with curative intent is ineffective and should only address complications. A surgical biopsy can be performed only with diagnosis intent. The palliative surgery can be admitted in case of hemorrhage, external mass with high discomfort for the patient, stenosis, or obstruction of the anal canal [14]. Nevertheless, survival does not exceed 1 year from diagnosis. Together, the present and published cases indicate that perianal masses may be misdiagnosed and patients with NSCLC or other cancers should be evaluated for metastatic disease.

**Table 1:** Major characteristics of patients reported in the literature

Year	Journal	First author	Age	Sex	Primary	Metastases	Presentation	Syn or Met	Sin or Mul	Treatment	Survival
1968	Dis Colon Rectum [3]	Ger R	49	M	Squamous cell carcinoma (NSCLC)	Heart; anus	Rectal hemorrhage	Syn	Mul	Abdominoperineal resection	Third postoperative day.
1975	Dis Colon Rectum [4]	Kanhouwa S	45	M	Anaplastic large-cell carcinoma (NSCLC)	Mediastinum; anus	Anal ulcer	Met	Mul	CCNU and hydroxyurea; radiotherapy	10 months
1988	Gan No Rinsho [5]	Uenura Y	78	M	Mucoepidermoid carcinoma (NSCLC)	Multiple (nr); anus	Perianal mass	Syn	Mul	Radiotherapy	<1 year
1994	Surg Today [6]	Kawahara K	75	M	Squamous cell carcinoma (NSCLC)	Atrium; anus	Anal polyp	Met	Mul	Transanal polypectomy	nr
2006	Gastroenterolo Clin Biol [7]	Wisniewski B	53	M	Adenocarcinoma (NSCLC)	Anus; mediastinum; brain	Anal abscess	Met	Mul	Incision and drainage	1 month
2007	Southern Medical Journal [8]	Tek I	50	M	Adenocarcinoma (NSCLC)	Anus	Anal abscess	Met	Sin	Chemotherapy; drainage of abscess	nr
2010	J Buon [1]	Okutur K	64	M	Squamous cell carcinoma (NSCLC)	Pleura; anus	Anal polyp	Met	Mul	Excision of the polyp; radiotherapy	nr
2013	Clin Resec Hepat Gastroent[10]	Camus M	53	F	Adenocarcinoma (NSCLC)	Liver; mediastinum; anus	Perianal ulceration	Syn	Mul	Chemotherapy	nr
2014	Inter Medic [11]	Imai H	36	F	Adenocarcinoma (NSCLC)	Subcutaneous; anus	Perianal mass	Met	Mul	Resection of perianal mass	nr
2015	Int J Colorectal Dis [12]	Guerra F	75	F	Small cell lung cancer (SCLC)	Anus	Perianal abscess	Met	Sin	Chemotherapy	nr
2015	Present study	Al Tarakji M	75	M	Adenocarcinoma (NSCLC)	Bone; anus	Perianal abscess	Met	Mul	Supportive care	1 month

*Syn* synchronous, *Met* metachronous, *Sin* single, *Mul* multiple, *nr* not reported

## Conclusions

Because perianal masses may be misdiagnosed, patients with lung and other cancers should be evaluated for metastatic disease.

## Abbreviations

*NSCLC* non-small cell lung cancer, *CT* computed tomography, *PET* positron emission tomography
